# Water management improvement in PEM fuel cells via addition of PDMS or APTES polymers to the catalyst layer

**DOI:** 10.3906/kim-2002-49

**Published:** 2020-10-26

**Authors:** Hande UNGAN, Ayşe BAYRAKÇEKEN YURTCAN

**Affiliations:** 1 Faculty of Engineering, Department of Chemical Engineering, Atatürk University, Erzurum Turkey; 2 Graduate School of Science, Department of Nanoscience and Nanoengineering, Atatürk University, Erzurum Turkey

**Keywords:** PEM fuel cell, water management, PDMS, APTES, hydrophobicity

## Abstract

Water management is one of the obstacles in the development and commercialization of proton exchange membrane fuel cells (PEMFCs). Sufficient humidification of the membrane directly affects the PEM fuel cell performance. Therefore, 2 different hydrophobic polymers, polydimethylsiloxane (PDMS) and (3-Aminopropyl) triethoxysilane (APTES), were tested at different percentages (5, 10, and 20 wt.%) in the catalyst layer. The solution was loaded onto the surface of a 25 BC gas diffusion layer (GDL) via the spraying method. The performance of the obtained fuel cells was compared with the performance of the commercial catalyst. Characterizations of each surface, including different amounts of PDMS and APTES, were performed via scanning electron microscopy (SEM) and energy dispersive X-ray spectroscopy (EDX) analyses. Molecular bond characterization was examined via Fourier transform infrared spectroscopy (FTIR) analysis and surface hydrophobicity was measured via contact angle measurements. The performance of the fuel cells was evaluated at the PEM fuel cell test station and the 2 hydrophobic polymers were compared. Surfaces containing APTES were found to be more hydrophobic. Fuel cells with PDMS performed better when compared to those with APTES. Fuel cells with 5wt.% APTES with a current density of 321.31 mA/cm ^2^ and power density of 0.191 W/cm ^2^ , and 10wt.% PDMS with a current density of 344.52 mA/cm ^2^ and power density of 0.205 W/cm ^2^ were the best performing fuel cells at 0.6V.

## 1. Introduction

Recently, green energy has become the lifeblood of society and the economy. Negative effects, such as environmental pollution, deterioration of the ecological balance, and the effect of greenhouse gases, have led scientists to search for alternative, renewable, low-carbon emission energies.Proton exchange membrane fuel cells (PEMFCs), which have the most basic use in hydrogen energy systems, are considered to be a very promising technology for converting chemical energy into electrical energy[1,2]. In the case of using hydrogen as fuel, only water and heat are produced as an outcome product, meanwhile zero or probably very low carbon emissions might be obtained. Moreover, harmful emissions, such as nitrogen dioxide, sulfur dioxide, or carbon monoxide, would not pass on [3]. PEM fuel cells are environmentally friendly power sources with low emissions and operating temperatures, and high energy efficiency [3–5]. Fuel cells are quiet technology because they do not have moving parts, so they are ideal for use in mobile applications, hospitals, mobile phones, and laptops [6].

Structurally, in PEM fuel cells, the membrane electrode assembly (MEA) is placed between the 2 gas diffusion layers (GDLs), which stand on the catalyst layers where both reduction and oxidation reactions takes place. Hydrogen gas is fed into the anode electrode and oxygen gas or air flow through the cathode electrode. As a result of the electrochemical reaction occurring in the catalyst layers of these electrodes, water and heat are released. The function of the proton exchange membrane, which is impermeable against the reactant gases, is to allow the hydrogen protons to transfer from the anode electrode to the cathode electrode.

Hydrogen is the simplest element, consisting of an electron and a proton. The hydrogen gas that is fed into the anode electrode in the PEM fuel cell is divided into electrons and protons in the catalyst layer of this electrode, which is called an oxidation reaction. While the protons move (travel) through the membrane and pass into the cathode electrode, the electrons are transferred via an external circuit to the cathode side.Protons and electrons combine with oxygen fed into the cathode electrode and produce the water molecule, which is called a reduction reaction [7].Thus, while heat is released as a result of reactions occurring in the catalyst layers of the PEM fuel cell, water is produced in the cathode electrode.

One of the biggest factors that hinder the commercialization of PEM fuel cells is the lack of proper water management [8].For this reason, water management is crucial to improve the performance of PEM fuel cells. It has been recognized in recent studies that the wettability of GDL surfaces is critical to keep fuel cells fully viable at high-current densities [9]. The water molecules in the membrane structure form weak hydrogen bonds with the protons and conduct the transmission of protons by electro-osmotic drag from the anode to the cathode side, so the membrane needs to be saturated with water. In other words, proton transmission is directly related to membrane humidity. If the membrane is not hydrated adequately, an increase in heat and ohmic losses is also observed due to the increment in electrical resistance[10].

The opposite of this issue occurs in cathode electrodes. The low operating temperature of the PEM fuel cell causes water to condensate, and electro-osmotic drag that allows the protons to pass through the membrane occurs from the anode side to the cathode side, and water is also produced as a result of the reduction reactions in the cathode catalyst layer. For these reasons, there is excess water on the cathode side, called water flooding, which prevents the reactants from accessing the catalyst active sites, thus significantly reducing fuel cell performance, because the water droplets coming out of the cathode GDL gradually grow and block the gas channels [11].Even liquid water can flow in channels as film or slug flow regime, depending on flow conditions. The gas flow rate can be increased to remove this excess water from the channel, but this causes power loss in the fuel cell [8]. It is believed that the flooding event at the cathode electrode causes mass or concentration losses[12].

In PEMFCs, optimum water management is one of the most critical points to prevent flooding, minimize mass transport or concentration losses, and increase performance. It is the task of the GDL to remove heat and excess water, prevent the clogging of pores, and establish a balance between the membrane humidity and flooding phenomena[13,14]. For this reason, the GDL is quite an important PEMFC component for water management. Hydrophobic polymers, such as polytetrafluoroethylene (PTFE) can be used in the GDL to contribute to water management [15–17]. Since the water is produced on the cathode side, the use of a hydrophobic agent is very important, especially in the cathode electrode. The use of PTFE as a hydrophobic polymer is quite common in the literature[16–18]. Nguyen et al. prepared a hydrophobic catalyst solution using PTFE nanoparticles to ensure proper transport of the gas and liquid phase [19]. Friedmann and Nguyen investigated the contents of the catalyst components in their research and reported that the ink composition of Nafion:Teflon:C=1.375:0.375:1 had the highest activity [20,21]. Avcioglu et al. reported that when using PTFE polymer in the catalyst layer, the cell performance increased from 0.28 W/cm ^2^ with Pt/C-nafion solution to 0.32 W/cm ^2^ with the addition of 30% PTFE at 0.45 V [22]. However, the amount of PTFE has a significant effect on fuel cell performance, as electrical conductivity decreases due to an increasing amount of PTFE. The optimum PTFE proportion that should be used is between 10% and 30% [15,16,18,23]. Cabasso et al. prepared a 50–300-µm-thick GDL surface with a catalyst ink containing poly (vinylidene fluoride) and used it in PEM fuel cells[24].Lim et al *.* determined the temperature-changing contact angles and optimum amount of fluorinated ethylene propylene (FEP) carbon papers prepared by impregnating FEP. They showed that the contact angle decreased with increasing temperature and obtained a higher power density for 10% FEP-impregnated MEA than for 30% FEP-impregnated MEA [16]. Salahuddin et al. produced GDL surfaces using carbonized polyacrylonitrile nanofibers. The surface hydrophobicity was adjusted using the superhydrophobic agent (PTFE). Water condensation tests showed that using a superhydrophobic substance contributed significantly to water management[25].

This paper offers innovative polymers for PEM fuel cells that will contribute to water management, and thus improve fuel cell performance. Polymers containing methyl groups show hydrophobic character. These polymers do not degrade easily with heat, water, chemicals, or oxidizing agents. They are known as long-lasting polymers because of their resistance to moisture and sunlight. Another impressive characteristic of silicones is that they have high gas permeability in thin film layers [26]. These polymers with hydrophobic character have excellent properties, such as low surface energy, high surface activity and gas permeability, and easy workability.

Polydimethylsiloxane (PDMS) and (3-Aminopropyl) triethoxysilane (APTES) are polymers with hydrophobic properties with the methyl group. PDMS is a silicon-type polymer. Silicon polymers have a long life span and are resistant to heat and oxidizing agents[26]. Due to its high surface activity, good workability, high gas permeability, and transparency, PDMS has a wide range of uses. APTES is a frequently preferred polymer due to its unique surface properties[27]. It is an aminosilane often used in surface functionalization [28]. Aminosilanes facilitate the formation of siloxane bonds, and amine groups enable them to have catalytic activity[29].

PDMS is a polymer that has been used in previous studies and achieved good results [30]. A different GDL (25 BC) was chosen in this study. The results of this study were compared with a previous study. APTES is a polymer that has never been used before in hydrophobic catalyst studies in fuel cells. The polymers presented in this study are highly promising for developing GDL surfaces with higher performance and good water management in PEM fuel cell applications in the future.

## 2. Experimental

### 2.1. Materials

A commercial platinum/carbon (Pt/C, 66.7% w/w Pt; Tanaka) catalyst, Nafion solution, (15% w/w; Ion Power Inc., New Castle, DE, USA) was used to ensure proton conductivity, and as a hydrophobic agent, PDMS (236.53 g/mol; Sigma-Aldrich Corp., St. Louis, MO, USA), APTES (221.37 g/mol; Sigma-Aldrich Corp.),as a solvent,2-propanol (99.5% Sigma-Aldrich Corp.), and pure water were used to prepare a catalyst solution. GDL 25 BC (Sigracet GDL) and Nafion (NR-212, Ion Power Inc.) were purchased as the GDLs and membrane, respectively.

### 2.2. Catalyst preparation

Electrochemical reactions in PEM fuel cells are catalyzed by a platinum catalyst. Therefore, both the anode and cathode solutions contained Pt. The main purpose of this study was to control the water management on the cathode electrode side. Therefore, PDMS and APTES, which are hydrophobic polymers, were added in different amounts only to the catalyst solutions prepared for the cathode electrode. The catalyst inks prepared for the anode electrode consisted of 70% Pt/C and 30% Nafion ionomers. For this reason, the commercial catalyst, which was calculated as 0.4 mg/cm ^2^ Pt per area of the GDL, and Nafion ionomer, were dissolved in 4 mL of 2-propanol and 2 mL of distilled water as a solvent, and mixed in a magnetic stirrer for 24 h at room temperature. The prepared inks were mixed using an UltraTurrax homogenizer (Wilmington, NC, USA) for 5 min and the catalyst was dispersed well in the solution. The catalyst inks prepared for the cathode electrode also contained 70 wt.% Pt/C (0.4 mg/cm ^2^ Pt per area of the GDL). In order to investigate the effect of the amount of polymer on fuel cell performance, different amounts (5, 10, and 20 wt.%) of polymer were added to each ink. The amount of Nafion ionomer was calculated according to the amount of polymer used. The catalyst inks consisted of 30 wt.% Nafion ionomers + hydrophobic polymer. In Table 1, the chemicals and amounts in the inks prepared for the catalyst layer are given. H-0 represents only the solution prepared with the commercial catalyst (without PDMS or APTES) for the anode electrode side. The inks prepared by adding 5, 10, and 20 wt.% PDMS were named HP-5, HP-10, and HP-20, while the inks prepared by adding 5, 10, and 20 wt.% APTES were named HA-5, HA-10, and HA-20, respectively (Table 1).

**Table 1:** Amount of chemicals in the catalyst inks.

Catalyst	Pt/C (mg)	Nafion (mg)	PDMS (mg)	APTES (mg)
H-0	2.65	1.15	---	---
HP-5	2.65	0.95	0.19	---
HP-10	2.65	0.76	0.38	---
HP-20	2.65	0.38	0.76	---
HA-5	2.65	0.95	---	0.19
HA-10	2.65	0.76	---	0.38
HA-20	2.65	0.38	---	0.76

### 2.3. MEA preparation

**Figure 1:**
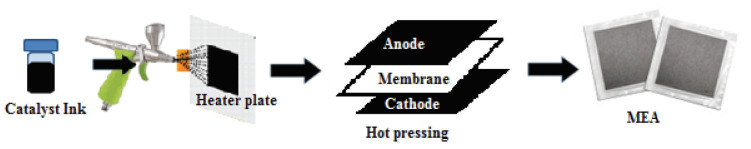
MEA preparation steps.

The inks were loaded onto GDL (Sigracet, 25 BC GDLs) surfaces with an area of 4.41 cm ^2^ by spraying them onto a vacuum heater plate. MEA structures were formed by combining the anode, membrane, and cathode trio (triplet). The anode electrode prepared only with commercial catalyst was the same in every MEA. Each cathode electrode was prepared according to the chemical contents given in Table 1. MEA preparation conditions, such as the temperature, pressure, and time, were very important for the best adherence of the catalyst layer loaded on the GDL. The highest temperature the membrane could withstand was 135°C, while optimization for the compression pressure was achieved at 400 psi and 4 min, and an untreated polymer electrolyte membrane was used. Between these 2 electrodes, polymer electrolyte membrane Nafion 212 was placed and pressed at 130 °C and 400 psi for 4 min so that the MEA was obtained. The MEA preparation steps are shown in Figure 1.

### 2.4. Physical characterization

Fourier transform infrared spectroscopy (FTIR) is used to characterize material with absorption peaks corresponding to the frequency formed by the vibration between atoms of organic or inorganic molecules. Since the type, shape, and size of the atoms are effective in the formation of these absorption peaks, spectroscopic methods provide quantitative and qualitative analysis. The materials have their own spectrum, like a fingerprint. It is possible to make molecular bond characterization and have information about functional groups with FTIR analysis. In this study, spectroscopic analysis of the electrode surfaces was performed at room temperature in the range of 400–4000 cm ^-1^ using a Bruker Vertex 70V model FTIR analyzer (Bruker Corp., Billerica, MA, USA).

The scanning electron microscopy (SEM) analysis, which provides important information in 2 dimensions, is very important for the morphological and microstructure characterization of the catalyst layer on the GDL surface. SEM provides the opportunity to measure the distribution of elements on the surface when used together with energy dispersive X-ray spectroscopy (EDX) analysis. Thus, the presence of elements such as F, Si, and N indicated that the desired polymers were present on the surface. In this study, a Zeiss Sigma 300 field emission SEM instrument (Carl Zeiss Microscopy GmbH, Oberkochen, Germany) was used for the SEM analysis of the GDL surfaces.

The static contact angle, defined as the angle formed at the interfaces of solid, liquid, or gas phases, is a method often used in determining the hydrophobic or hydrophilic characterization of a surface. The static contact angle is measured by dropping 5 µL of test liquid (water is generally preferred in fuel cell applications) onto the GDL surface in a circle and measuring the external angle with a tangent line from where the liquid droplet touches the surface. Static contact angles of the surfaces were measured using a Biolin Scientific attension theta t330 model device (Biolin Scientific AB, Göteborg, Sweden).

### 2.5. Fuel cell testing system

The fuel cell performances of the MEAs using APTES and PDMS hydrophobic polymers to improve water management in PEM fuel cells were measured using a Henatech ^TM^ single test station (Henatech, Turkey). The MEAs were located between 2 silicon gaskets (125µm) for sealing and placed in the middle of the single test cell. The anode and cathode, 2 parts of the cells, were combined and compressed by applying 2.5 Nm of torque force to the screws. First, nitrogen gas at 0.1 L/min was sent to purify the system from impurities until the system temperature reached thermal stability at 70 °C.

After the system reached the hydrodynamic equilibrium, nitrogen gas delivery was stopped, and hydrogen gas was sent to the anode electrode side, while oxygen gas was sent to the cathode electrode side, at a rate of 0.25 L/min. The gases were humidified at the desired temperature using humidifier tanks that were controlled by a proportional integral derivative temperature controller before they came to the cell, because, as is known, humidification is a very important factor in ensuring the transmission of protons through the membrane. The humidified reactive gases allowed the membrane to be moistened, and thus assisted in proton transfer.

Fuel cell performances of each MEA were measured at gas humidification temperatures of 50, 60, and 70°C. Although the humidification temperatures of the gases changed, the fuel cell temperature was kept constant at 70 °C during the measurements. While the system was stable and humidification conditions were provided, the electronic load was opened, which was connected to the system and reflected the voltage current values to the computer screen. The first read current value was called the open circuit voltage (OCV). Starting the OCV value at 0.1 V, the voltage was reduced at 0.05-V intervals and current densities corresponding to this voltage were recorded. Recording of the performance was repeated every half hour until the current densities were constant. In the half-hour period between the 2 performance readings, the cell was held at 0.6 V. The obtained voltage-current densities were graphed and interpreted.

## 3. Results and discussion

**Figure 2:**
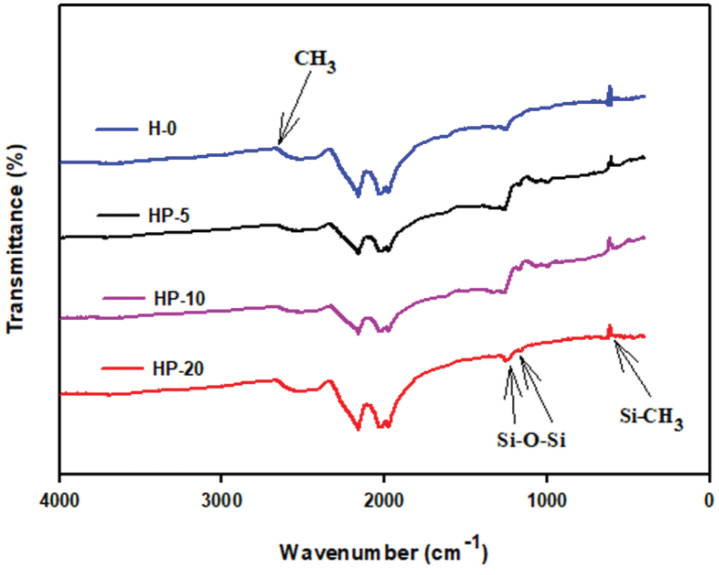
FTIR spectrum of the GDL surfaces containing different amounts of PDMS and Tanaka.

Molecular bond characterization was examined and functional groups of the polymers were identified by performing FTIR analysis of the GDL 25 BC surfaces loaded with different amounts of the corresponding polymers. Figure 2 shows the FTIR spectrum of the prepared GDL 25 BC surfaces loaded with H-0, HP-5, HP-10, and HP-20. The band of CH _3_ groups seen at 2906 cm ^1^ was attributed to symmetrical stretching. The peaks observed at 1258 and 1410 cm ^-1^ , especially on the HP-5 and HP-10 surfaces, represented symmetrical and asymmetric bending vibrations of the CH _3_ groups, respectively. The symmetrical and asymmetrical stretching vibrations of Si-O-Si were observed at 1072 and 1007 cm ^-1^ , respectively. The band at 864 cm ^-1^ was characteristic of Si-CH _3_ group asymmetric rocking [31–33].

**Figure 3:**
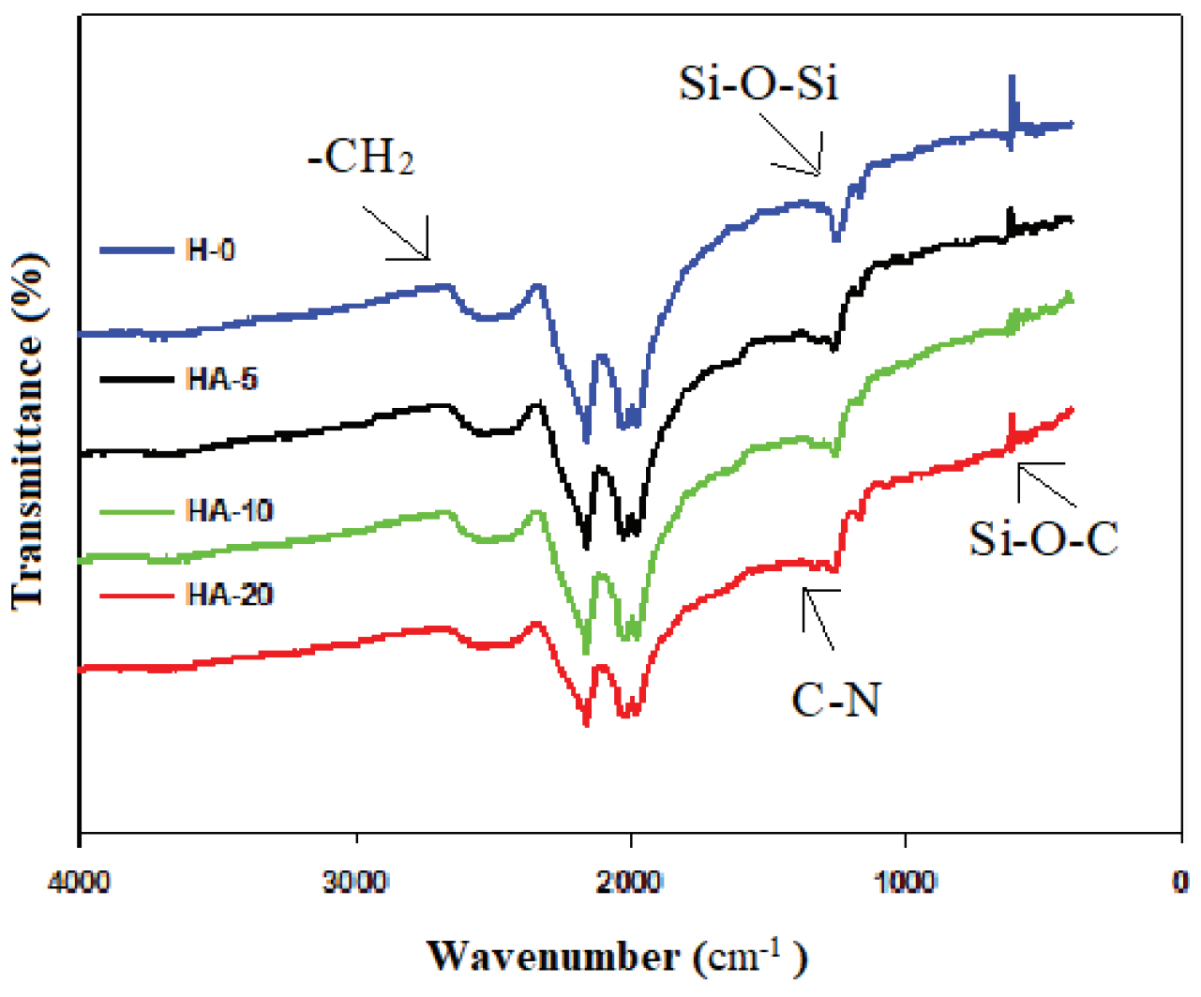
FTIR spectrum of the GDL surfaces containing different amounts of APTES and Tanaka.

Figure 3 shows the FTIR spectrum for the H-0, HA-5, HA-10, and HA-20 surfaces. The peak points of characteristic Si-O-Si at 1050 cm ^-1^ , Si-O-C at 780 cm ^-1^ , -CH _2_ at 2890 cm ^-1^ , and -NH at 1630 cm ^-1^ , belonging to the APTES polymer, were observed in the spectrum [34,35]. The bands at 2852 and 2932 cm ^-1^ can be attributed to the C-H bending vibrations of the APTES. Specifically, the C-N peaksat 1302 cm ^-1^ and C=N peaksat 1610 cm–1were observed quite intensely on the surface of HA-20 [35].

**Figure 4:**
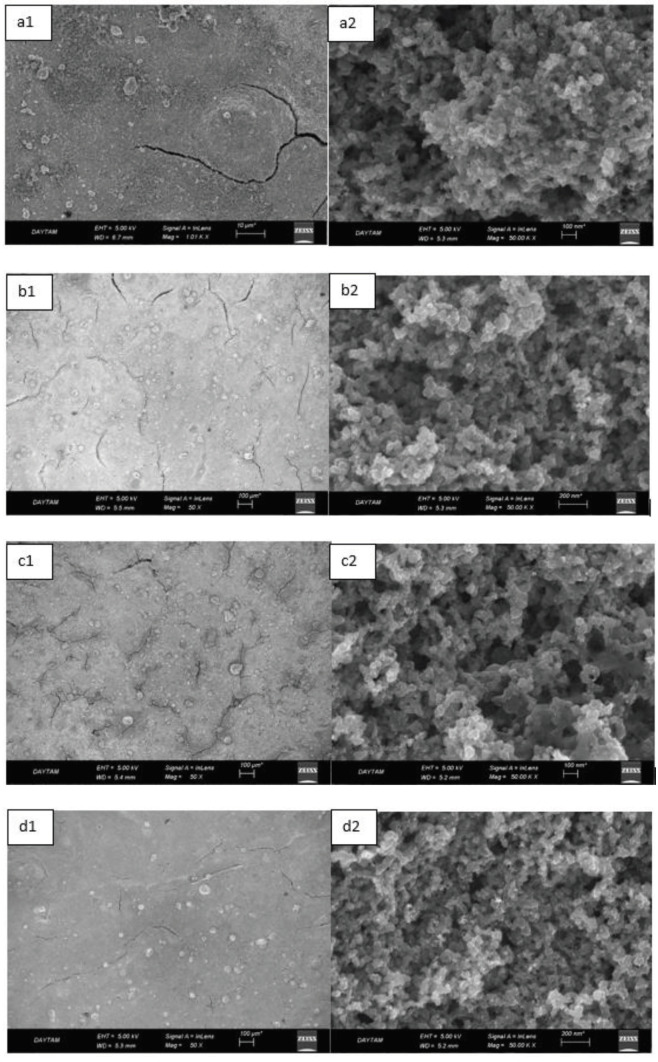
Scanning electron microscopy images of the H-0 (a1-a2), HP-5 (b1-b2), HP-10 (c1-c2), and HP-20 (d1-d2) GDL surfaces with 50,000-fold magnification (right side) and 50-fold magnification (left side).

Figure 4 shows the SEM images of the H-0, HP-5, HP-10, and HP-20 GDL surfaces. Obtaining a homogeneous surface coating is very important for fuel cell performance. High thermal zones are formed due to increasing electron and proton permeability resistance in a region where homogeneous coating cannot be achieved. The increasing heat might cause regional temperature increases wherein structures called pinholes are formed on the surface. This situation negatively affects the performance. The crack and macro-sized holes can especiallybe seen in Figures 4a1, 4b1, 4c1, and 4d1 . A more compact dispersed carbon powder/hydrophobic polymer structure was obtained by the increasing polymer content (see Figure 4d1). Uniform dispersion of the solution on the electrode surface provided a decrease in contact resistance. Thus, electrical conductivity increased [36].

**Figure 5:**
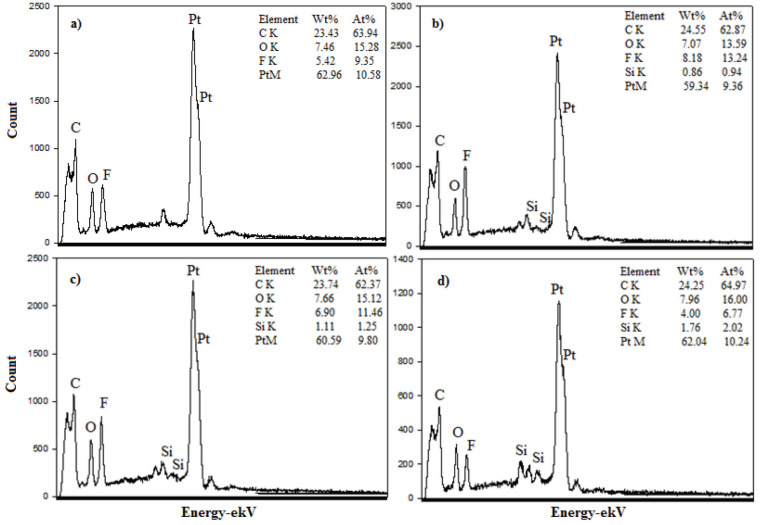
EDX spectra of the H-0 (a), HP-5 (b), HP-10(c), and HP-20 (d) GDL surfaces.

Figures 5a–5d show the EDX analysis results for the H-0, HP-5, HP-10, and HP-20 GDL surfaces. The carbon (C) acquired from the analysis resulted from both the carbon paper in the GDL content and the carbon black in catalyst content. Fluorine (F) was caused by both the Nafion ionomer and the PTFE polymer contained in the GDL. Platinum (Pt) refered to the commercial catalyst content loaded on the electrode by spraying. Silicon (Si) obtained for the HP-5, HP-10, and HP-20 was a result of silane groups in the structure of the PDMS polymer.The results of the analysis proved that the catalyst inks were successfully loaded onto GDL surfaces. The presence of Si in the EDX analysis increased due to the increase in the amount of PDMS.

**Figure 6:**
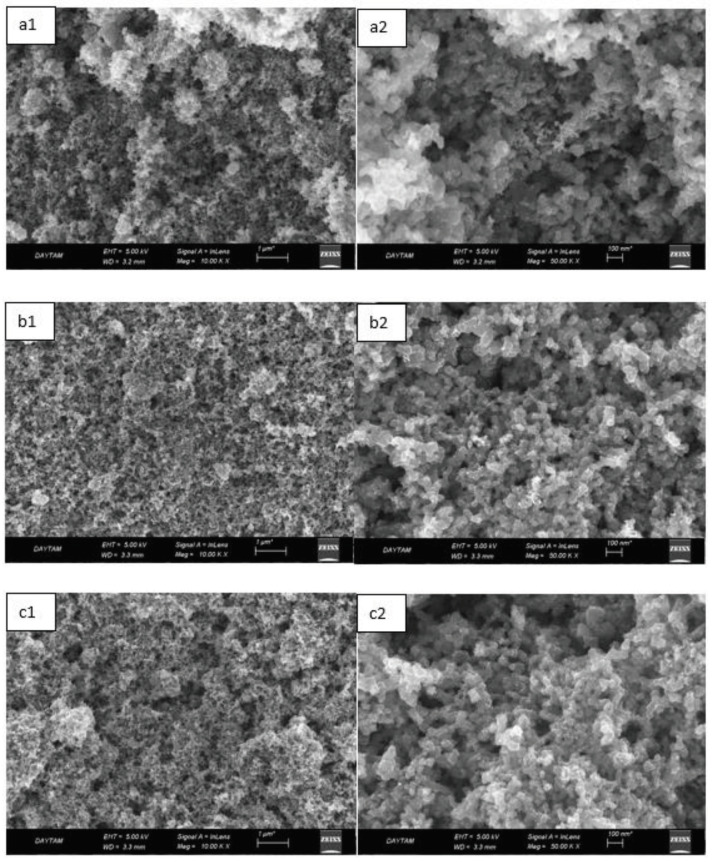
Scanning electron microscopy images of the HA-5 (a1-a2), HA-10 (b1-b2), and HA-20 (c1-c2) GDL surfaces with 50,000-fold magnification (right side) and 10,000-fold magnification (left side).

In Figure 6, SEM images of HP-5, HP-10 and HP-20 GDL surfaces are shown. The texture, crystal structure, and orientation of the obtained GDL surfaces were determined by SEM analysis. It can be seen that the GDL surface provided homogeneous distribution. Only Figures 6a1 and 6a2 show agglomeration of the ionomers along the surface when compared to Figures 6b1–6c2. Compact, homogeneous, and porous surface morphology is highly desirable for GDL coatings. The dark regions that are particularly visible in Figures 6c1 and 6c2 represent nano-sized pores and were thought to facilitate mass transfer[37]. Furthermore, as the amount of polymer increased, it was clear that the gap between the carbon structures filled and agglomeration decreased.

**Figure 7:**
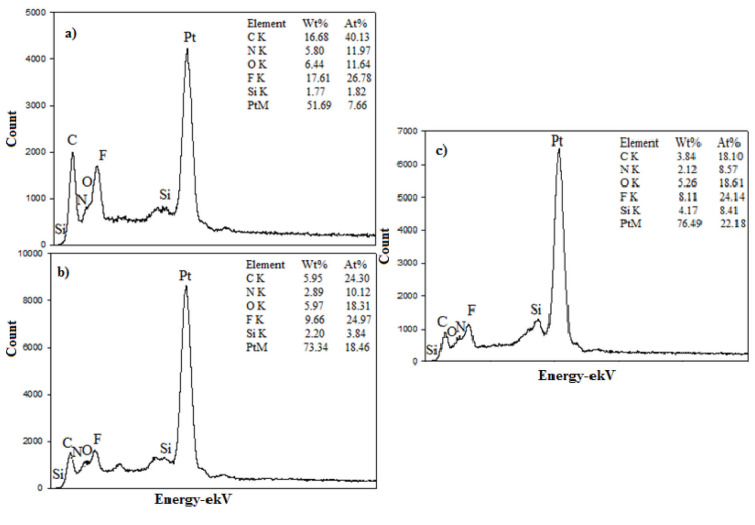
EDXS spectra of the HA-5 (a), HA-10 (b), and HA-20 GDL (c) surfaces.

Figures 7a–7c show the EDX analysis results for the HP-5, HP-10, and HP-20 surfaces. EDX analysis provides insight into elemental composition. C, N, O, F, Si, and Pt obtained on the surfaces as a result of the analysis proved that the catalyst inks were successfully loaded. Nitrogen (N) obtained for HA-5, HA-10, and HA-20 was the result of amine groups in the structure of the APTES polymer. The precursors of the other elements were the same as described in Figure 5.

The contact angle is a quantitative measure of how a solid surface is wetted with a liquid. The contact angle between a solid surface and a liquid can be between 0 and 180°. The magnitude of the contact angle depends on the distribution of the liquid on the solid surface. The longer the fluid stays intact, the greater the angle. For a perfect wetting, the contact angle must be 0°; in this case, the liquid is spread over the solid surface as a thin film. If the angle is less than 90° the liquid can be said to wet the surface, if it is greater than 90° it does not wet the surface. According to this, if the angle between the solid and liquid is less than 90°, these surfaces are called hydrophilic surfaces, whereas 90° or more of these surfaces are called hydrophobic surfaces. Surfaces with contact angles higher than 150°are also called superhydrophobic surfaces. On hydrophobic surfaces, the water drop does not spread to the surface and takes a spherical shape. On hydrophilic surfaces, however, it cannot maintain drop form and disperses to the surface [38].

The importance of water management in PEM fuel cells is related to the reach of reactive gases to the GDL surface.If excessive amounts of water accumulate on surfaces, this causes the surfaces to be blocked and prevents the reactants from reaching and diffusing on the GDL. Consequently, this negatively affects fuel cell performance. For this reason, GDL surfaces should be both hydrophobic enough to prevent excessive water accumulation and moist enough to ensure conductivity. In this study, contact angle measurements of the GDL surfaces prepared by loading catalyst inks containing different percentages of hydrophobic polymers (APTES or PDMS) were taken and compared with each other in terms of their hydrophobic characteristics. Contact angles of all of the prepared electrode surfaces at room temperature, and 50, 60, and 70 °C (PEM fuel cell operating temperature) were measured.

Table 2 indicates the contact angle measurement results for the H-0, HP-5, HP-10, and HP-20 surfaces. The surface contact angles for the H-0, HP-5, HP-10, and HP-20 surfaces were obtained at room temperature as 152.60°, 157.77°, 158.99°, and 161.84°, respectively. The contact angles of all of the surfaces loaded with the catalyst ink were above 150 °C and showed a superhydrophobic character. The results at 50 °C were measured as 137.31°, 148.39°, 154.90° and 155.16°, respectively. The surface contact angles were measured for each operating temperature to correlate the contact angle with the temperature. The contact angel values at 60 °C were measured as 132.12°, 145.27°, 145.48°, and 150.84°, respectively, and at 70 °C were measured as 119.62°, 136.38°, 139.25°, and 140.52°, respectively. The increasing amount of PDMS added to the GDL surface caused the surface contact angles to gradually grow. The HP-20 surface, which contained 20% polymer, had the highest contact angle for each measurement at different temperatures. It was clearly seen that the temperature hada significant effect on the surface morphology and hydrophobic character. Increasing the temperature reduced the surface hydrophobicity. The contact angle decreased as the temperature increased.

**Table 2:** Contact angles of the H-0, HP-5, HP-10, and HP-20 GDL surfaces at room temperature, and 50, 60, and 70°C.

Temperature	H-0	SamplesHP-5	HP-10	HP-20
Room temperature	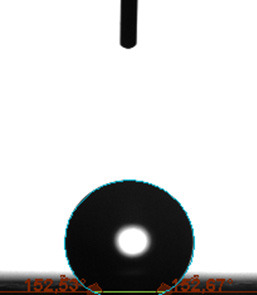	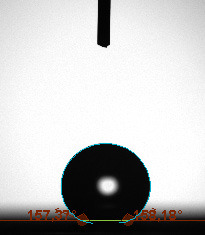	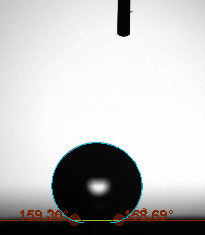	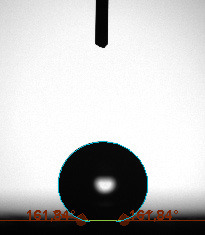
50 °C	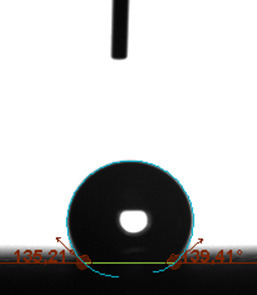	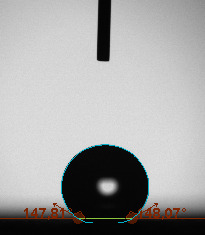	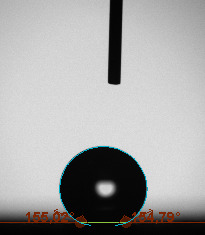	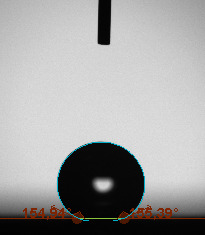
60 °C	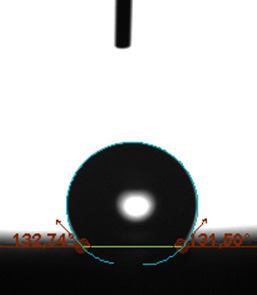	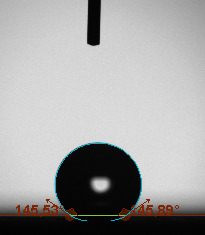	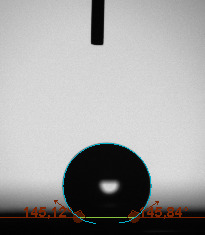	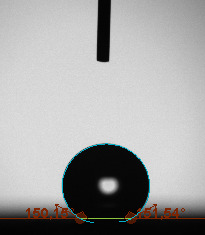
70 °C	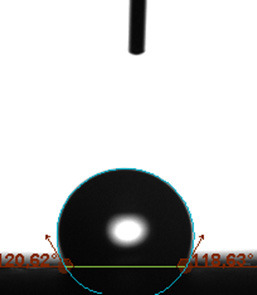	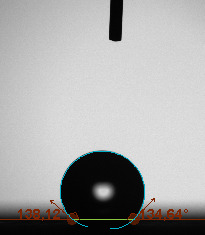	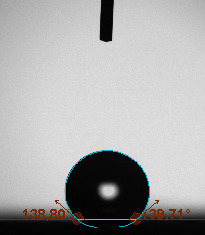	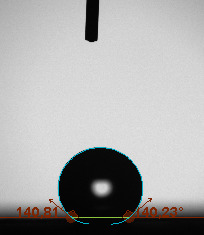

Table 3 shows the contact angle measurement results for the HA-5, HA-10, and HA-20 surfaces. The surface contact angles for the HA-5, HA-10, and HA-20 surfaces were obtained at room temperature as 157.62°, 162.35°, and 168.49°, respectively. At 50 and 60 °C,the results were measured as 140.64°, 157.41°, and 158.31°, and 140.15°, 147.19°, 151.15°, respectively. Contact angle values decreased as the temperature increased and for 70 °C, the results were measured as 121.44°, 145.03°, and 149.34°, respectively. The contact angles of the GDL surfaces prepared with the APTES polymer were higher than those prepared with the PDMS polymer. The contact angle is a measure of the wettability of a surface. The fact that polymers can be connected to water with hydrogen bonds is characteristic of its hydrophobic property. In this study, different amounts of polymers were loaded on the GDL surface and the fuel cell was operated at different temperatures. These measured contact angles for different quantities and temperatures showed that as the amount of polymer on the surfaces increased, the surface hydrophobicity increased. A decrease in contact angle values was observed with increasing temperature.

**Table 3:** Contact angles of the HA-5, HA-10, and HA-20 GDL surfaces at room temperature, and 50, 60, and 70°C.

Temperature	HA-5	SamplesHA-10	HA-20
Room temperature	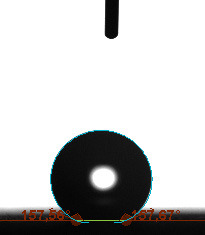	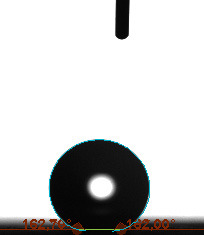	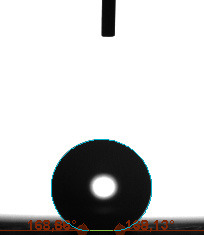
50 °C	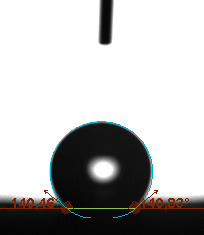	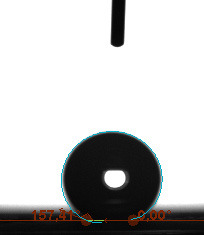	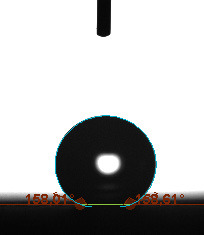
60 °C	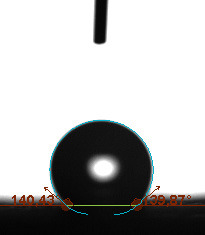	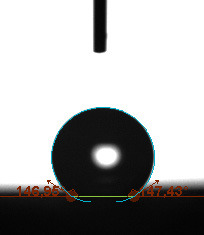	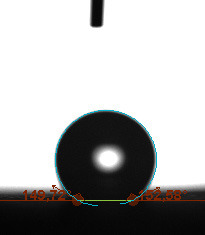
70 °C	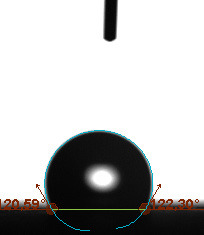	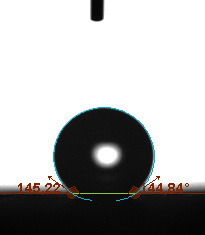	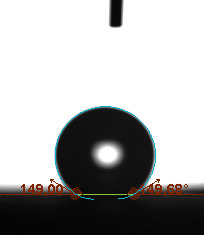

**Figure 8:**
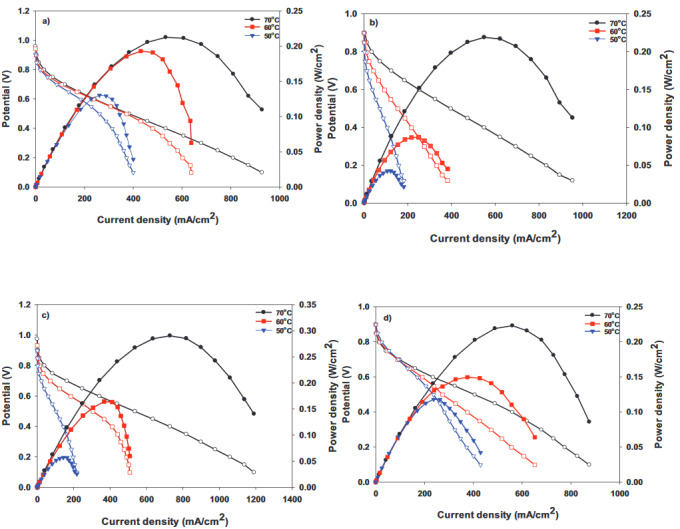
Polarization curves of the samples: (a) H-0, (b) HP-5, (c) HP-10, and (d) HP-20 at 50, 60, and 70 °C.

**Figure 9:**
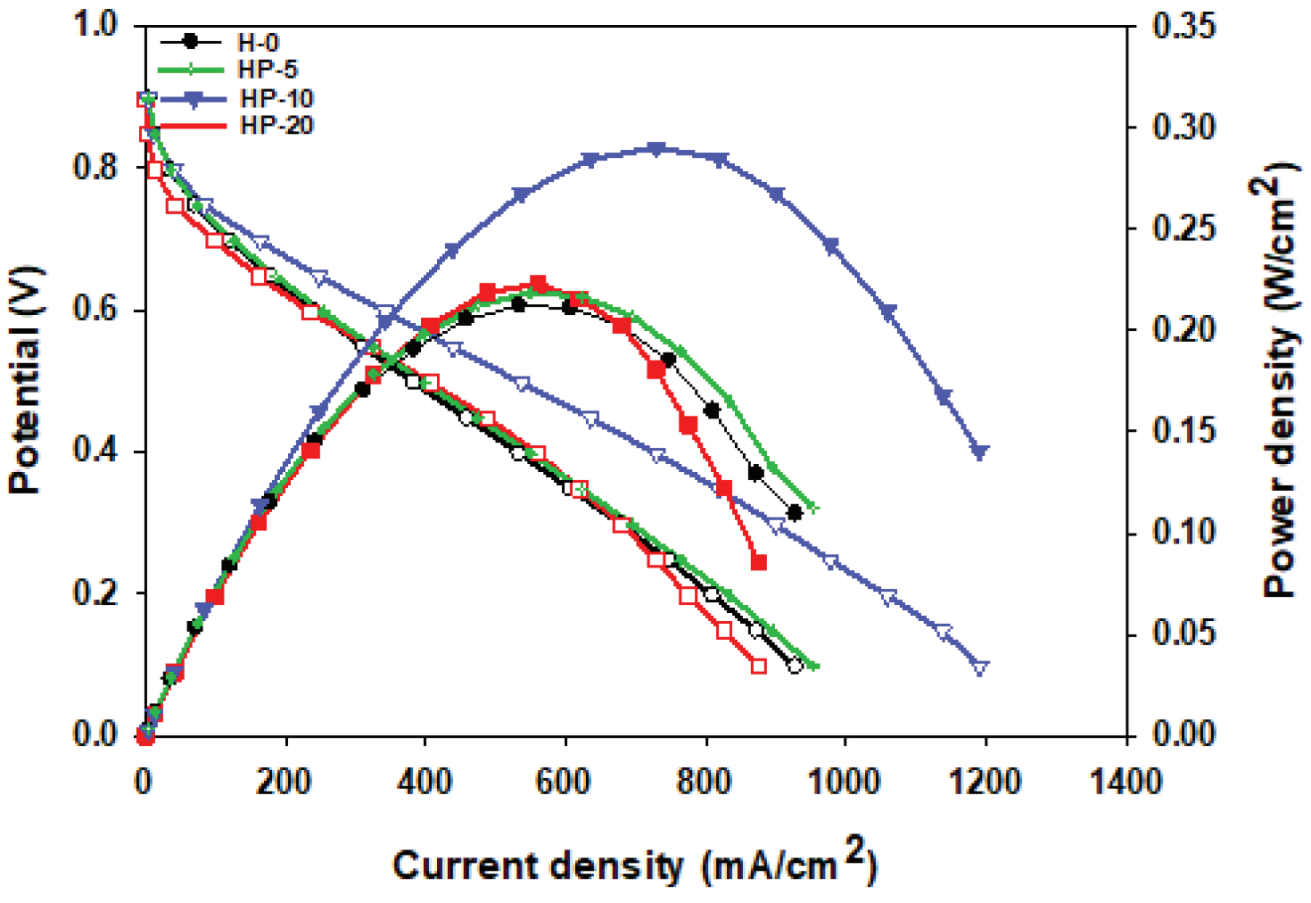
Comparison of the polarization curves for HP-5, HP-10, and HP-20 (containing PDMS) with H-0 (Tanaka) at 70 °C.

Fuel cell performance analysis of each of the MEAs acquired by placing a conducting Nafion membrane between 2 electrodes, prepared by adding different amounts of hydrophobic polymer to the cathode side, was achieved at 50, 60, and 70 °C. Figures 8a–8d show the polarization curves at 50, 60, and 70°C for the H-0, HP-5, HP-10, and HP-20 surfaces.Since 50 °C is a low temperature in terms of fuel cell performance, power densities from all of the cells were very low at this temperature. Hence, the best cell performance was achieved at 70 °C, and this temperature was preferred when comparing cells containing different amounts of PDMS, as shown in Figure 9. The current densities obtained at 70 °C for the H-0, HP-5, HP-10, and HP-20 surfaces at 0.6V were 242.63, 253.74, 344.52, and 234.92 mA/cm ^2^ , respectively. The best performance belonged to the HP-10 MEA. This result showed that the addition of 10% PDMS was sufficient to provide optimum water management for fuel cell performance. In a previous study, catalyst inks with 5, 10, and 20 wt.%. PDMS were loaded onto the surface of a different type of GDL (GDL 34 BC, Sigracet) [30]. The results obtained in this study were lower than the performance results obtained for the MEA structures prepared using GDL 34 BC as the GDL in the previous study. Radev et al. found similar results for MEAs prepared on GDL 25 BC surfaces using a Tanaka catalyst to examine the effect of membrane thickness on performance and cell durability [39]. The reason for the lower performance of the GDL 25 BC, which has a thinner (235µm) and more porous structure[39], was the increased contact resistance caused by the catalyst ink loading thickness [40].

**Figure 10:**
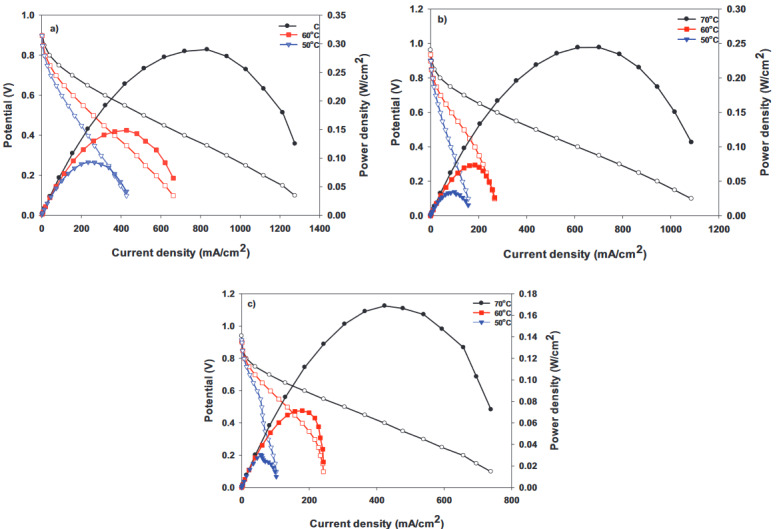
Polarization curves of the samples: (a) HA-5, (b) HA-10, and (c) HA-20 at 50, 60, and 70 °C.

Figures 10a–10c show the polarization curves at 50, 60, and 70 °C for the HA-5, HA-10, and HA-20 surfaces. The contact angle measurement results showed that the APTES polymer-loaded surfaces were more hydrophobic. One of the important parameters for cell performance is sufficient humidity, so it is important to find the optimum amount of polymer that keeps the membrane humid enough, and allows proton transition and prevents flooding.

**Figure 11:**
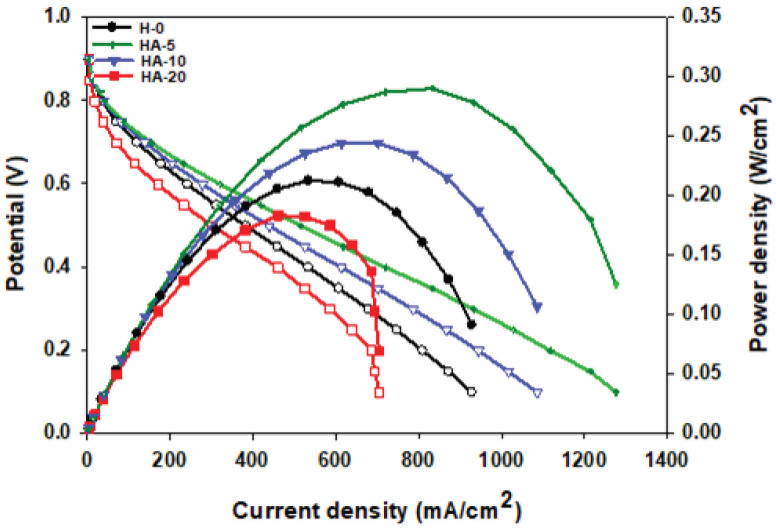
Comparison of the polarization curves for HA-5, HA-10, and HA-20 (containing APTES) with H-0 (Tanaka) at 70 °C.

Martin et al. obtained electrodes by using different amounts of Pt electrodeposited on a commercial carbon cloth, forming a 5–10 µm-thick layer of carbon black. The single cell test results of the electrodes were found to be between 50 and 550 mA/cm ^2^ at 0.6 V [41]. Martin et al. loaded different amounts of Pt on unhydrophobic carbon papers (without PTFE) via the electrospray method. The performance of these cells, prepared without using hydrophobic polymer, was quite low and was measured as 100 and 400 mA/cm ^2^ [42]. Avcioglu et al. reported that the cell performance they obtained by adding 5, 10, 20, and 30 wt.% PTFE was 76, 64, 35, and 33 mA/cm ^2^ , respectively. They argued that as the PTFE amount increased, the Pt and carbon structures were isolated by PTFE nanoparticles, thereby reducing Pt use and decreasing performance [43]. Performance tests for different humidification temperatures showed that the MEAs prepared were unsuitable for the operation at 50 °C. The power density taken from the fuel cell was very low at this temperature. Polarization curves of MEAs containing different amounts of APTES are shown in Figure11. The current densities obtained at 70 °C for the HA-5, HA-10, and HA-20 surfaces at 0.6Vwere 321.31, 278.45, and 170.97 mA/cm ^2^ , respectively. The best cell performance was achieved for the HA-5 surface. The polymer added to the catalyst ink for the HA-20 surface gave the membrane excessive hydrophobicity, causing a decrease in fuel cell performance.

## 4. Conclusions

In order for fuel cells to be commercialized and disseminated, restrictions on their use need to be eliminated. Water management is one of these limitations and almost the most important. In this study, 2 new polymers that contributed to water management were conducted as alternatives to the hydrophobic polymers traditionally used in PEM fuel cells. New hydrophobic surfaces were developed for the PEM fuel cell. PDMS polymer was used in a catalyst ink on a different surface (GDL 34 BC) than in previous studies and good results were obtained. The APTES polymer has not been used previously in the PEM fuel cell catalyst layer and was used for the first time herein.The surfaces were prepared using different amounts of 2 different hydrophobic polymers (PDMS and APTES) and physical analyses of these surfaces were performed.Contact angle measurements showed that adding polymer dramatically increased surface hydrophobicity. The contact angle incredibly exceeded as the amount of polymer added increased.In addition, by changing the operating conditions, each prepared fuel cell was tested at the fuel cell station under humidity conditions of 50, 60 and 70 °C, and 70 °C was found to be the optimal temperature for the fuel cell operating temperature. The decrease in temperature led to a severe reduction in cell performance. The best fuel cell performance was obtained for the surface containing 10% PDMS at 70°C.Surfaces containing both APTES and PDMS performed better than the commercial catalyst. This indicated that adding hydrophobic polymer to the catalyst ink exceptionally improved the surface conditions. The results showed that although the cell containing 10% PDMS polymer gave the best performance, the APTES polymer exhibited a greater stable condition when compared with the PDMS polymer at high current densities. This study also showed that different types of polymers have different optimum amounts for the best performance of fuel cells.
